# Supplementary figures and images for: Investigation of NLR Genes Reveals Divergent Evolution on NLRome in Diploid and Polyploid Species in Genus Trifolium
Source: Genes (Basel). 2023 Apr 4;14(4):867. doi: 10.3390/genes14040867 (PMC10138078; doi:10.3390/genes14040867)

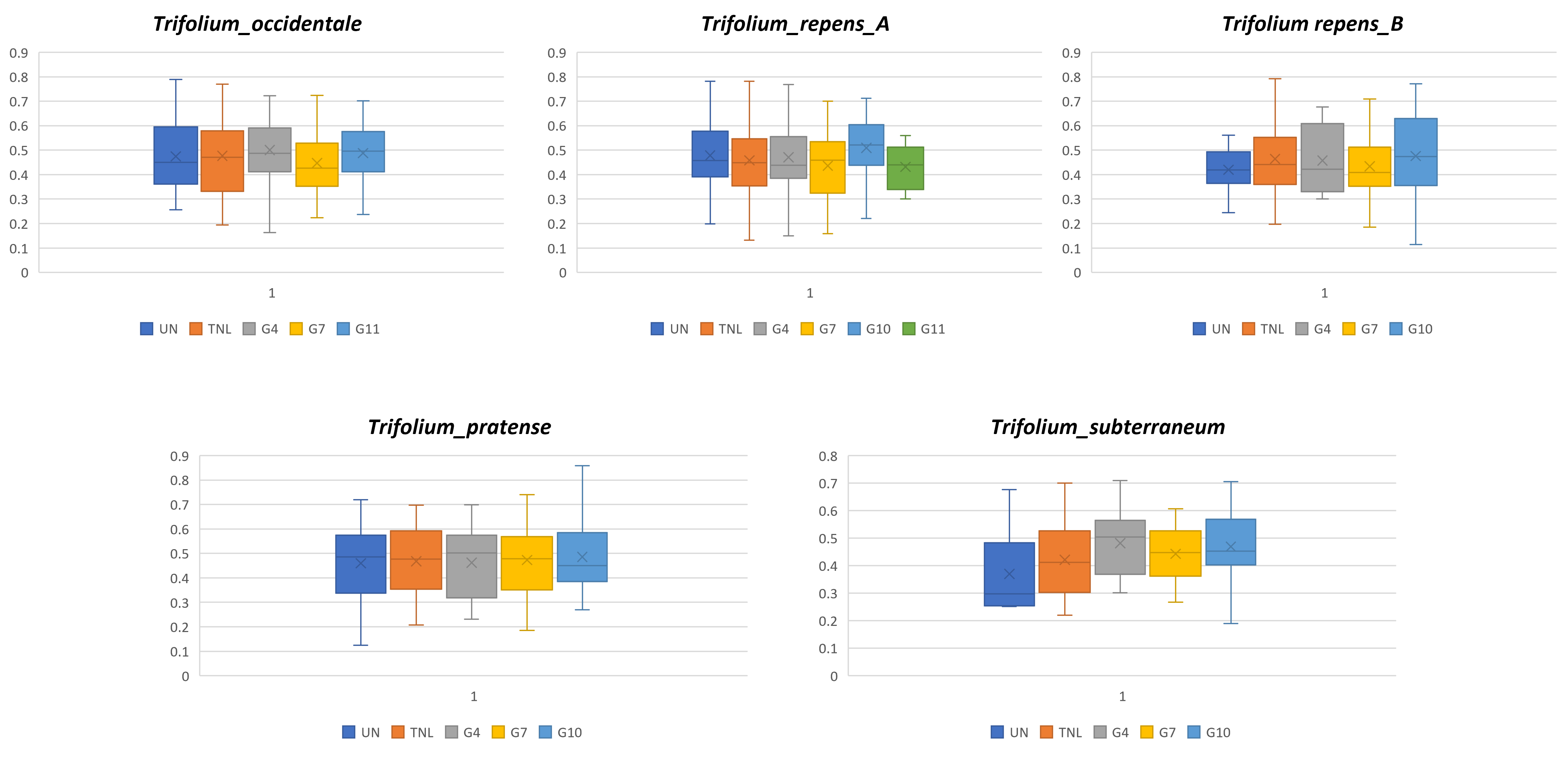

Supplement: Supplementary file 1 [file genes-14-00867-s001.zip › Figure S1 Kakas_subgroups.png]

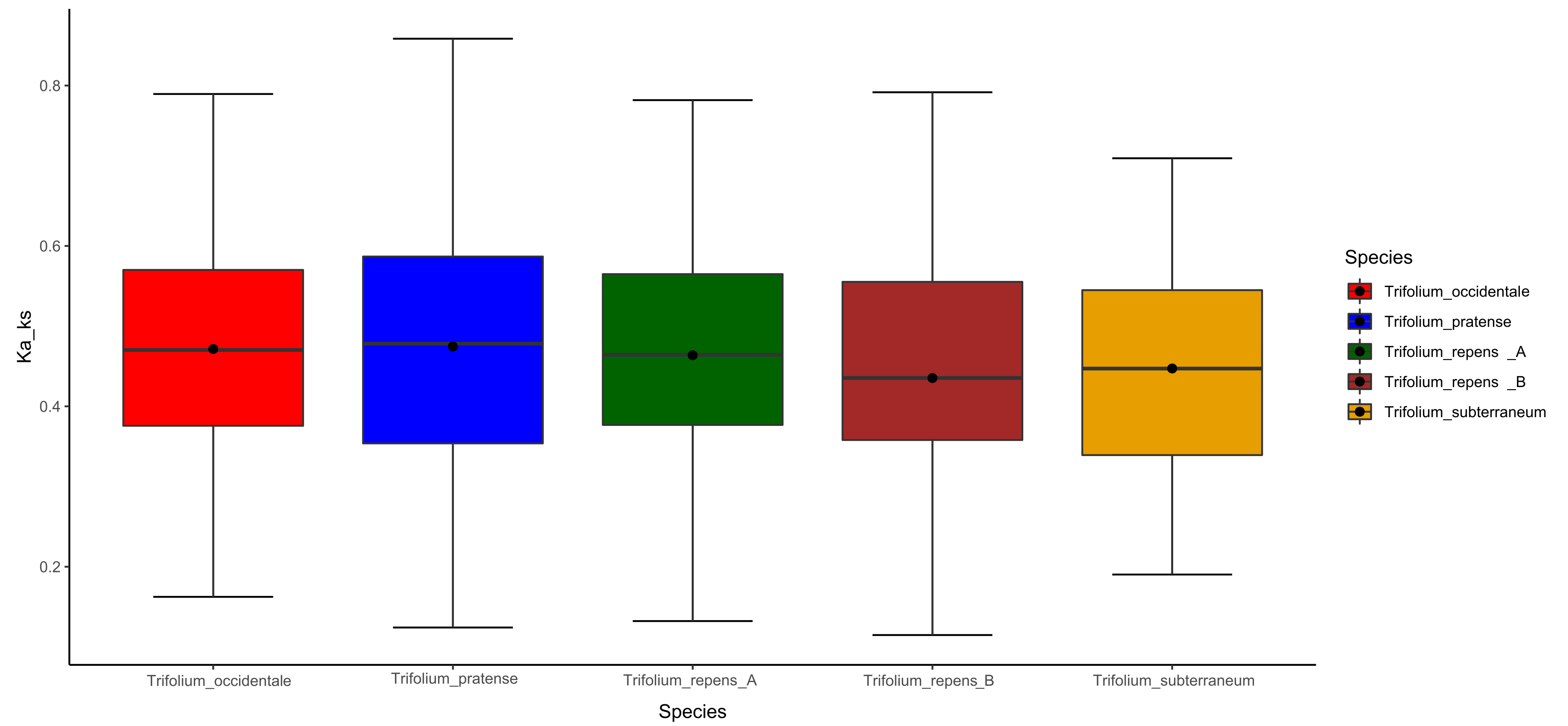

Supplement: Supplementary file 1 [file genes-14-00867-s001.zip › Figure S2.png]

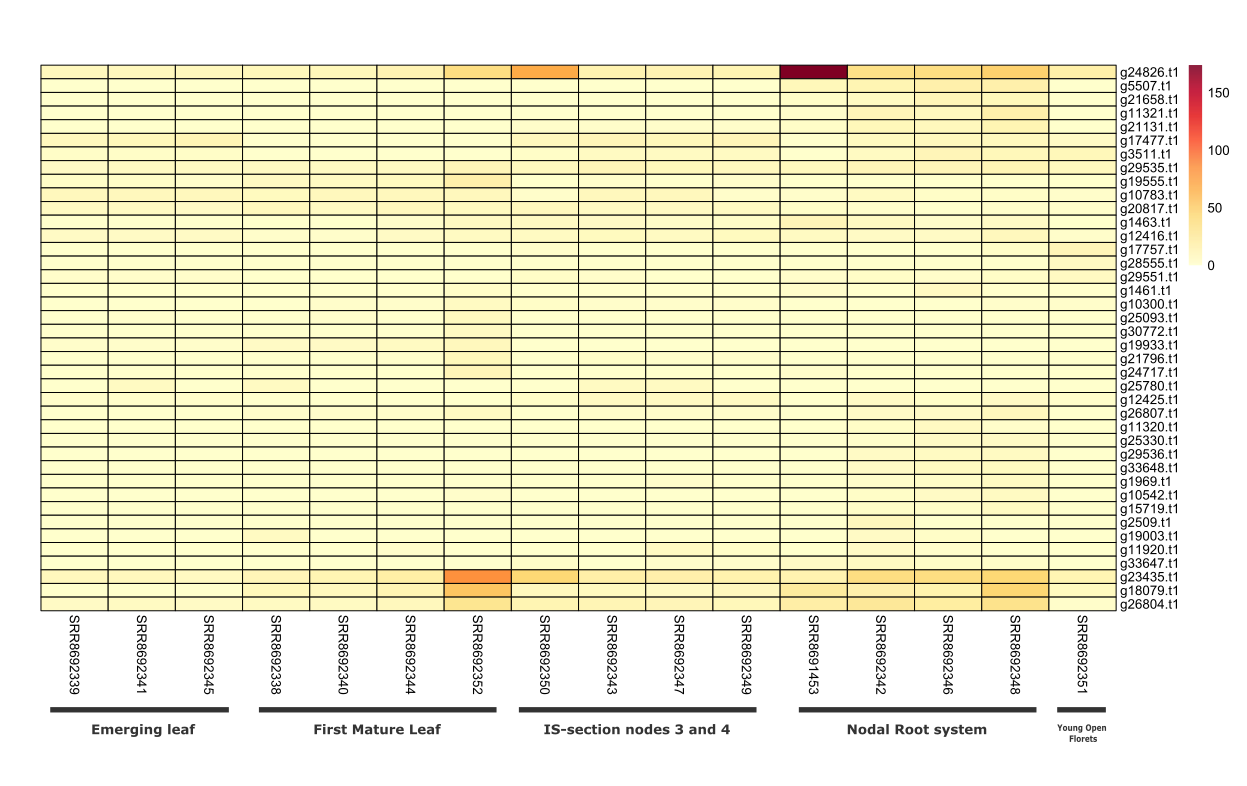

Supplement: Supplementary file 1 [file genes-14-00867-s001.zip › Figure S3.png]
